# Development of an AFASS assessment and screening tool towards the prevention of mother-to-child HIV transmission (PMTCT) in sub-Saharan Africa - A Delphi survey

**DOI:** 10.1186/1471-2458-12-402

**Published:** 2012-06-06

**Authors:** Stella M Adegbehingbe, Virginia Paul-Ebhohimhen, Debbie Marais

**Affiliations:** 1 Division of Applied Health Sciences, University of Aberdeen, Aberdeen, AB25 2ZD, UK; 2 Health Services Research Unit, University of Aberdeen, Aberdeen, AB25 2ZD, UK

## Abstract

**Background:**

The rate of mother-to-child transmission of HIV, occurring during pregnancy, delivery/labour and breastfeeding, still remains high in Sub-Saharan Africa (SSA). The World Health Organization recommends HIV infected mothers exclusively breastfeed their infants, unless replacement feeding is *Acceptable, Feasible, Affordable, Sustainable and Safe* (AFASS). Health care workers are responsible for providing counselling to mothers on the risks and benefits of infant feeding options allowing mothers to make an ‘informed choice’, but this role is challenging and mostly subjective. The aim of this study was to develop and content validate an AFASS assessment tool that could be used for infant feeding counselling in SSA.

**Methods:**

An AFASS assessment tool was developed based on the evidence and tools available regarding why replacement feeding is not AFASS in SSA (15 questions). Fifty seven experts involved in PMTCT programmes in five SSA countries were approached to participate as members of the Delphi expert panel (purposive sampling and snowballing). A web-based survey, utilising a 4-point Likert scale, was employed to gain consensus (>75% agreement) from the expert panel following the Delphi technique.

**Results:**

A final panel of 15 experts was obtained. Thirteen of the 15 questions in the tool achieved consensus agreement. Experts suggested some additional questions, and that double-barrelled questions were split. Consensus was achieved regarding the applicability and appropriateness of the tool within a SSA context. Experts all agreed that the tool will be useful for the purpose for which it was designed. Suggestions made by the expert panel were incorporated into the revised tool.

**Conclusions:**

The findings of this study confirm that this AFASS counselling tool may be appropriate and useful for SSA. Ideally the revised tool should be tested by providers of infant feeding advice with the aim of adoption into routine PMTCT programmes in SSA. Within the context of the 2010 WHO guidelines which advocate a public health rather than an individualised approach, it may inform the WHO process of improving counselling tools for health care workers involved in PMTCT programmes.

## Background

Human immunodeficiency virus/Acquired immunodeficiency syndrome (HIV/AIDS) remains a leading cause of death among children under five years in low/middle income countries. About 90% of children living with HIV reside in Sub-Saharan Africa (SSA) where, in the context of a high mortality rate, AIDS accounts for eight percent of all under-five deaths in the region [1,2]. In 2010, about 390 000 children under 15 became infected with HIV, mainly through mother-to-child transmission (MTCT) [3].

MTCT of HIV can occur either during pregnancy, labour and delivery (perinatal transmission) or through breastfeeding [4]. Where there is no form of treatment to prevent MTCT, 15-30% of babies born to HIV positive mothers become infected during pregnancy and delivery, and a further 20% will be infected through breastfeeding [5]. This risk can however be reduced to less than 2% where the appropriate treatment is used and the mother adheres to her choice of infant feeding [6] as has been achieved in high income countries [7].

The World Health Organisation’s (WHO) guideline for HIV and infant feeding in 2006 recommended that ‘HIV infected mothers should exclusively breastfeed (baby is fed with breast milk only, and no other liquid (including water) or solid with the exception of drops or syrups consisting of vitamins, mineral supplements or medicines is allowed) their infants for the first six months of life, unless exclusive replacement feeding is **AcceptableFeasibleAffordableSustainable** and **Safe** (AFASS)’ [8]. The underlying principle for this is still explicit in the 2010 update by the WHO but national authorities are advocated to promote a public health approach rather than an individualised approach as counselling to support individualised decisions has not been implemented despite training and tools being provided [9]. Although this study was conducted before these guidelines were widely distributed, the results are still relevant and can inform the WHO process of improving counselling tools for health care workers involved in PMTCT programmes.

Mixed feeding (feeding both breast milk and foods or liquids) has been consistently proven to be associated with about a 2 to 10-fold increase in the risk of MTCT during the first six months of life compared to exclusive breastfeeding [10-12]. In Africa, the practice of mixed feeding, often from a very young age [13] has been observed to be a major obstacle to the implementation of this guideline [14-16]. Furthermore, exclusive replacement feeding which can reduce the risk of HIV transmission is not often AFASS in low income countries [17,18] due to factors including increased risk of death from diarrhoea and malnutrition [19], the high cost of artificial formula [20], and stigma associated with not breastfeeding [21].

Current evidence supports a key principle that the choice of infant feeding cannot be generalized for all HIV infected mothers as there are qualitative and quantitative factors that influence choice and actual feeding practice [20,22-24]. A decision on infant feeding for children born to HIV infected mothers should therefore be tailored and based on careful consideration of the HIV mother’s individual and other life circumstances to achieve maximum compliance to whatever choice is made, thus reducing the likelihood of mixed feeding.

The counselling role of health workers in guiding HIV infected women to make an informed choice of feeding option is challenging. Balancing the risks and benefits of infant feeding choices is difficult and training counsellors to understand the complexities of feeding practices takes time and dedicated supervision. As much as infant feeding counsellors want to base their guidance on knowledge and judgement, this could be an emotive decision with some counsellors unintentionally influencing mothers’ decision [25,26]*.* A qualitative study involving nurse counsellors in prevention of MTCT (PMTCT) sites in Tanzania also reported that counsellors experienced stress and frustration, felt inadequate in their ability to counsel mothers, and that patients tended not to trust them. This study recommended the need for urgent training and provision of support as an essential requirement for achieving the desired goal [27].

This study was conducted towards developing a locally relevant, objective and standardised tool for assessing mothers’ circumstances when providing counselling on available infant feeding options in SSA, which could be easily administered by health care workers in counselling mothers on infant feeding options thereby supporting mothers in making an informed choice.

## Methods

An observational descriptive study design, using quantitative measurement was employed in this Delphi methods survey. Following an extensive literature review to explore reasons why replacement feeding is not AFASS in SSA, and collation of information from some established algorithms [24,28] and counselling cards by the WHO and UNICEF [29], a AFASS assessment tool was developed.

**The AFASS tool** was divided into two sections. Questions (Table 1) in the first section (preliminary section) aimed to assess mothers’ knowledge about the risk and benefits of breastfeeding and replacement feeding. A 'yes' response to all the questions in this section was considered an essential prerequisite to proceeding to the next section, while a 'no' response to any of the preliminary questions, was considered as a flag for the need for an initial discussion with the mother, to explain the risks and benefits of each infant feeding option.

**Table 1:** Agreement AFASS Tool and Consensus

**Question**	**Agreement with the Question**	**Agreement with the language**
**Preliminary questions**		
Do you know you can pass HIV on to your baby through breastfeeding?	13 (87)	
Do you know your baby is more likely to get diarrhoea, pneumonia or malnutrition when you don’t prepare formula feeds correctly or hygienically?	13 (87)	
Do you know that the risk of transmitting HIV to your baby is three times higher when you mix feed (i.e. breastfeeding and formula feeds) than breastfeeding only?	11 (73)	
Do you know you stand the chance of getting pregnant soon if you choose not to breastfeed or use contraceptive?	13 (87)	
**Acceptability questions**		
Would you be able to carry on with your choice of feeding (breastfeeding or formula feeding) at home, in that you would not conform to others’ expectations?	14 (94)	7 (47)
Does the father of your child or other people close to you know your HIV status?	15 (100)	14 (94)
If you choose to formula feed, would you be comfortable giving infant formula in public/community?	15 (100)	13 (87)
**Feasibility questions**		
Can you manage to prepare and feed your baby every 2–4 hours day and night for up to three months?	12 (80)	11(73)
Will you be home for the first six months and not be working full time?	7 (47)	7 (47)
Do you have anybody assisting you with the care of your baby?	14 (93)	11 (73)
Do you have clean running water in your home/compound or close by?	14 (93)	12 (80)
Do you have a refrigerator in your home?	13 (87)	12 (80)
Do you have constant supply of electricity?	14 (93)	11 (73)
Would you be able to buy formula milk anytime it is required within your neighbourhood?	14 (93)	10 (67)
**Affordability questions**		
Can you afford $...... (Amount varies for different countries) on infant formula, utensils and cooking fuel for at least six months without disrupting the health and nutrition of other family members?	15 (100)	15 (100)
Do you have a source of regular income?	14 (93)	12 (80)
**Safety questions**		
Would you be able to wash your hands after using the toilet and before preparing feeds, as well as wash and sterilize all utensils required for preparing the feeds?	12 (80)	12 (80)
Do you have a water borne latrine/flush toilet in your home?	11 (73)	11 (73)
Do you have easy access to a health centre that provides Maternal and Child Health services?	15 (100)	13 (87)

The second section (AFASS section) comprised fifteen questions as follows: three questions relating to ***Acceptability*** of the AFASS criteria, seven questions to ***Feasibility***, two questions to ***Affordability*** and three questions to ***Safety***. The ***Sustainability*** aspect of the AFASS criteria was considered as interwoven in all other four criteria, hence no specific section for ***Sustainability*** was required.

Each question was designed such that the mother can answer ‘yes’ or ‘no’, and a ‘yes’ is in favour of replacement feeding. A total of fifteen responses was possible and the total number of ‘yes’ responses to be used to classify the recommended feeding option i.e. a score higher than 12 would favour replacement feeding as the preferred feeding option, a score less than or equal to 8 would favour breastfeeding as the preferred feeding option, while a score between 9 and 12 would identify a need for further counselling and discussion to determine the best option with the mother.

**The Delphi survey and data collection:** Experts for the purpose of this study were defined as health care workers who provide counselling or are involved in policy or decision-making in anti-retroviral clinics for pregnant and nursing mothers in SSA countries. Purposive sampling of experts involved in PMTCT programmes in five English speaking SSA countries (Nigeria, South Africa, Malawi, Kenya and Namibia) where investigators had direct contacts was conducted. Additional recruitment via snowballing was incorporated to increase representativeness of the sample. Forty-three experts were initially invited via e-mail to participate in the study as part of an expert panel. In the e-mail, the purpose of the research was explained and a request for participants to nominate other known colleagues involved in PMTCT programmes in their or other SSA countries was made. An expert panel of 10–15 members was expected given the relative homogeneity of the panel, in being constituted of participants (experts) living or working in similar geographical areas, and the subject of the study being focused [30].

The tool was sent to experts who consented to participate by e-mailing a link to the web-based questionnaire, and sought their opinion on usefulness, appropriateness and applicability of the tool in view of content validation and consensus generation. The web-based questionnaire was developed using the Snap survey tool (Snap 8 Professional). A 4-point Likert scale of agreement was used, ranging from strongly disagree to strongly agree. Questions were based on the items in both sections of the tool and experts were asked to indicate their agreement of whether the item should be included in the final tool. For the AFASS section, experts were also asked whether they agreed that the language/words used for each item was appropriate for SSA and if not, to provide alternatives (open-ended question). Opinions were invited regarding the use of specific terminology (father, frequency of feeding, safe water and proximity of water) and the inclusion of cup-feeding using open-ended questions. Agreement with the cut-offs for classification of the tool results as well as usefulness, applicability and appropriateness in the SSA context of the tool overall was also determined using the 4-point Likert scale. The appropriateness of the length of the tool was determined using a 3-point Likert scale. Finally, experts were given the opportunity to provide any other suggestions or comments in an open-ended question.

Responses from all respondents were collated and analysed towards the development of a final tool. To achieve consensus, it was agreed *apriori* that a minimum of 75% of the experts had to agree (agree and strongly agree combined) with a question for inclusion in the final AFASS tool. In addition, general comments were synthesized and summarized together for consideration in the final AFASS tool. Questionnaire data were transferred to SPSS data analysis package and analysed by descriptive statistics.

The University of Aberdeen’s College of Life Sciences ethical review board (CERB) exempted the research from requiring ethics approval as the e-survey was completely anonymous and participation was voluntary.

## Results and discussion

The expert panel consisted of 15 participants who completed the e-survey out of the twenty-five who consented (representing a 60% response rate). The expert panellists were from five SSA countries (Nigeria, South Africa, Malawi, Kenya and Namibia) and a variety of professional groups including obstetricians (1), paediatricians (3), nutritionists (5), and HIV program and treatment officers (3) and university academics (3). Twenty-five of the 57 invited to participate (43 purposive sample and 14 through snowballing), consented to participate, but only 15 completed the Delphi process, out of which four (2 nutritionists, 1 HIV program and treatment officer and 1 university academics) where recruited through snowballing.

### Consensus agreement on the AFASS tool for feeding options

Consensus (>75% agreement = 12 or more) was reached for the questions enquiring whether the tool as a whole is appropriate, applicable and useful for SSA settings with 13, 12 and 12 experts agreeing respectively.

Table 1 shows the questions in the tool, number of experts agreeing and the percentage agreement obtained for each question. Three questions did not achieve consensus but one of these was still considered suitable for inclusion in the final tool based on synthesis of comments and review of current evidence. For all other questions, consensus was reached. Only questions where consensus was not reached or suggestions for improvement were made are discussed.

Only 11 of the experts agreed with the question in the section quantifying the increase in risk of HIV transmission associated. Twelve experts indicated that the risk of transmission associated with mixed feed is higher than the three-fold stated in the question.

In the acceptability section, seven experts disagreed with the appropriateness of the language used for the question assessing the ability of the mother to carry on with her choice of feeding. The majority (13) of the experts’ said the language was not appropriate as the question would be prone to misinterpretation, hence rephrasing before including the question in the tool was suggested. It should be noted that the tool was developed in English and it is clear that using the correct word is very important. This is something that will have to be taken into consideration when translating the tool into other languages as often a direct translation is not the best translation.

In the **feasibility** section, only 7 experts agreed with the question which enquired about the availability of the mother to be home for six months. The experts’ felt that a mother does not necessarily have to always be home with the baby before exclusive replacement feeding can be practised efficiently. It was argued that the question is more in support of exclusive breastfeeding, and even at that, six months is not feasible. It was further argued that a mother can express breast milk even if she is practising exclusive breastfeeding.

Other aspects of disagreement in the feasibility section related to the language or wording of the questions. The appropriateness of the language used to enquire whether the mother can prepare formula feed and feed her baby every 2–4 h, day and night for the first three months was supported by 11 experts. Most experts (13) suggested changing the three months to six months. They also pointed out the fact that the frequency of feeding will depend on the age of the baby. Rephrasing this question was suggested before including it into the final tool.

The appropriateness of the language used for the question assessing the availability of constant supply of electricity was supported by 11 experts. Summary of the experts’ opinion revealed misinterpretation of the question as 13 experts interpreted availability of constant electricity in relation to supply of cooking fuel for preparing formula feeds and suggested other local means of cooking fuel. This question had however been considered for inclusion in relation to preserving already prepared formula feed, indicating that rephrasing the question to make it clearer was needed.

The language used for the question assessing whether the mother would be able to buy formula milk any time it is required within her neighbourhood was supported by ten experts. Experts argued that mothers’ can obtain formula milk by other means and not necessarily within the neighbourhood. Hence, rephrasing the question to whether the mother can ensure a constant supply of formula feed at any time was suggested by 14 experts.

The appropriateness of the language used in enquiring about the availability of someone giving assistance with the care of the baby was supported by 12 experts. However, 11 experts suggested that the question should be expanded or made simpler by giving some examples of what is meant by ‘assisting in the care of the baby’, e.g. feeding, bathing, dressing and soothing and that emphasis should be placed on availability of assistance with the feeding of the baby.

Both questions in the **affordability** section, and the appropriateness of the language used in describing the questions achieved consensus, but the question enquiring whether the mother can afford the money to buy infant formula, utensils and cooking fuel required for at least six months was considered too complex by 11 experts. It was suggested that the question is broken down to a minimum of two questions, separating the availability of funds for purchasing the formula from the availability of funds for utensils and cooking fuel.

For the question enquiring whether the mother has a source of regular income, additional questions about whether the husband or father of the child has a source of regular income and the number of dependants was suggested by 12 experts.

Two out of the three **safety** questions assessing the safety of replacement feeding achieved consensus on both appropriateness of the question and of the language. However 14 experts suggested that the question enquiring whether the mother will be able to wash her hands after using the toilet and before preparing feeds, as well as sterilizing utensils should be split. It was suggested that ensuring washing of hands and keeping utensils clean are separate activities. Adding ‘clean water’ to the question was also suggested by seven experts, they argued that ensuring washing of hands and utensils must entail the use of clean water to achieve the required aim. Eleven of the experts agreed with the question enquiring about the availability of a water-borne latrine or flush toilet achieved. Four experts argued that measures to ensure clean hands and good hygiene after using the toilet was paramount rather than the type of sewage disposal system.

### General suggestions

The best way to describe the father of a child in SSA was enquired from the participants and 12 experts agreed on using father, as opposed to husband or partner.

The most appropriate and easily understandable means of describing how often a baby is fed was also enquired and seven experts agreed on the use of hourly rate. They said the use of number of times/day may result in the mother squeezing in feeding times for her convenience and not necessarily at the right time when the baby is supposed to be fed. Those that agreed with using the number of times/day argued that using an hourly rate might be difficult for mothers who have poor idea of time. The remaining two suggested using either of the two as appropriate, depending on the educational level of the mother.

Whether to use clean water or tap water to enquire about the availability of safe water that could be used for preparing formula feed was also debated upon. Nine experts argued that clean water is more appropriate as not all households have tap water, but still have access to clean water. The remaining six argued that tap water is appropriate as this is the standard for a source of clean water.

Proximity of water access was also deliberated, whether to use a specific distance to describe proximity or leave it relative by using the word ‘close by’. Eight of the experts argued that the use of a relative term is better, as some mothers do not appreciate spatial orientation or metric distance. The rest of the participants supported using a specific distance as it is more objective.

Whether to specify cup feeding as the appropriate way of giving formula feed was also enquired, with 12 experts agreeing that this should be included in the tool. A minority (3) however said it should not be included as this might confuse mothers since cup feeding is not widely used and there is no evidence that proves it is a safer means of feeding.

All experts suggested avoidance of double-barrelled questions e.g. Would you be able to wash your hands after using the toilet and before preparing feeds, as well as wash and sterilize all utensils required for preparing? They argued that one part of the question might be a positive answer while the other may not, hence affecting the overall conclusion. Two-thirds of the experts also suggested including the demographic data of the respondents at the beginning the tool.

### The cut-off points for feeding options

Only nine of experts agreed that the cut-off point for breastfeeding should be ≤8 (number of ‘yes’ responses). Also only 10 of experts agreed that the cut-off point for replacement feeding should be >12. Less than half (7) of the experts argued that a cut-off point of 12 for replacement feeding will make most women in SSA not eligible to practise exclusive replacement feeding.

### Proposed AFASS assessment and screening tool

Twelve out of the 15 questions in the tool clearly achieved consensus from the panel of experts and hence have been included in the final AFASS tool. Of the remaining three questions not achieving consensus, one was corrected and the other two excluded from the final tool. Further language changes and splitting of double-barrelled questions were incorporated as suggested by the panel of experts.

The question in the preliminary section was corrected as suggested by the panel of experts and supported by the literature. Experts pointed out that the risk of transmitting HIV through breastfeeding when mothers mix feed is higher than the conservative estimate of three-fold employed in the tool. Studies have reported that mixed feeding can lead to a 2 to 10-fold increase in the risk of HIV transmission from an HIV infected mother to her child during the first six months of life, compared to exclusive breastfeeding [10-12].

The question in the feasibility section that enquires whether the mother will be home for the first six months and not be working full time has not been included in the final AFASS tool, as this did not reach consensus agreement. Experts argued that to achieve an effective way of replacement feeding does not necessarily require the mother home all the time.

The question under the safety section relating to the availability of water-borne latrine or toilet has also been excluded from the final AFASS tool. Experts argued that measures after using the toilet are more important than the type of sewage system in the home, although a counter argument can be made that the type of sewage system available in a community reflects the risk of communicable diseases.

The aspect that needed the most attention was the cut-off points for decision-making guides to which feeding option would be most advisable under the individual circumstances.

During the course of this study, a new WHO guideline for infant feeding in low income countries which recommends an extensive anti-retroviral (ARV) regimen to either infant or mother and mixed feeding was released [9]. This is based on current evidence from recent studies that demonstrate that with ARV, MTCT can be reduced to around 2% and by extending a course of ARVs for either the mother or the infant, mixed feeding can be practised without the risk of transmission of HIV infection to the infant [31-33]. The WHO guideline still recommends that when ARV are not immediately available, the recommendations included in the 2006 HIV and infant feeding update (AFASS criteria) should provide useful guidance for mothers and health workers [34]. This shows that the continuing use of the AFASS criteria remains relevant particularly in low income countries where regular supply of ARV is still a significant challenge. For example, over half of the 1.4 million pregnant women living with HIV are estimated to have received antiretroviral drugs to prevent transmission of HIV to their infants. An estimated 53% [40–79%] of pregnant women living with HIV received anti-retrovirals to reduce the risk of transmitting HIV to their infants, up from 45% [37–57%] in 2008 and 15% [12–18%] in 2005. However, a large proportion continued to receive the less efficacious single-dose Nevirapine regimen [35]. It is also possible that the release of this guideline during the course of this study was a source of bias as this could have influenced some of the experts’ opinion.

Further possible bias might have arisen if respondents who consented to participate but did not complete the survey (making up two-fifths of initial respondents) were significantly different from those who completed the survey but an overview of location and professional group of respondents did not appear to suggest either of these two factors were significantly different.

## Conclusions

In reviewing the current evidence on why replacement feeding is not AFASS in SSA an AFASS assessment and screening tool that may be used by health care workers to assist in objective counselling and informed decision-making on infant feeding options was developed. This has been content validated as applicable to the SSA context. Within the context of the 2010 WHO guidelines which advocate a public health rather than an individualised approach, this study can inform the process of improving counselling tools for health care workers involved in PMTCT programmes [9], addressing previous challenges of one to one counselling. WHO also recommends heat-treating breast milk as a simple method for home pasteurizing breast milk that inactivates HIV while preserving milk’s nutritional and anti-infective properties. This has been proven by various studies [36-38]. However, this method is most successful among women who have disclosed their status, have supportive family members and believe in the efficacy of the flash-heating methods [39].

This study acknowledges the challenges of one to one counselling and in the spirit of evidence based public health practice, recommends that further assessment for face validity (the revised AFASS tool shown in Additional file 1, indicating questions and scoring should be tested by providers of infant feeding advice) is undertaken on the tool, ideally in a participatory and exploratory process, before routine use of the tool in a setting is locally agreed. The use of tool can also serve towards assuring that a chosen public health approach in a particular locality is evidence based. For example, the results of a survey of mothers using the tool may help to determine the proportions of women within each of the three final score areas, in a particular locality. This information may then be used to inform a public health policy, in which the chosen approach is the most effective, and cost efficient for the majority of population in that locality.

## Competing interests

The authors declare that they have no competing interests.

## Authors’ contributions

SMA participated in the design of the study, conducted the data collection & analysis and drafted the manuscript. DM conceived the study and participated in the design of the study. VAP participated in the design of the study. All authors read, critically revised and approved the final manuscript.

## Pre-publication history

The pre-publication history for this paper can be accessed here:

http://www.biomedcentral.com/1471-2458/12/402/prepub

## Supplementary Material

Additional file 1 Revised AFASS Tool. Click here for file
