# Supplementary material for: Development of an AFASS assessment and screening tool towards the prevention of mother-to-child HIV transmission (PMTCT) in sub-Saharan Africa - A Delphi survey
Source: BMC Public Health. 2012 Jun 6;12:402. doi: 10.1186/1471-2458-12-402 (PMC3441873; doi:10.1186/1471-2458-12-402)
Supplement: Additional file 1 — Revised AFASS Tool. [file 1471-2458-12-402-S1.pdf]

Case Identifier/Registration Number:

Name: Date of birth:

Address:

Preliminary questions

- **Do you know you can pass HIV on to your baby through breast feeding?**
- **Do you know your baby is more likely to get diarrhoea or malnutrition when you do not prepare formula feeds correctly or hygienically?**
- **Do you know that the risk of passing HIV on to your baby is ‘four times’ higher when you mix feed (i.e. breastfeeding and formula feeds) than breastfeeding only?**
- **Do you know that you stand the chance of getting pregnant soon if you choose not to breastfeed or use contraceptives?**

If the mother responds NO to any of the above questions, the health care worker/counsellor should discuss further with the mother.

**AFASS:** **A**CCEPTABLE~**F**EASIBLE~**A**FFORDABLE~**S**USTAINABLE~**S**AFE		**YES**	**NO**
1	Would you be able to carry on with your choice of feeding (breastfeeding or formula feeding) even in the presence of pressure to change from family and friends?		
2	Does the father of your child know your HIV status?		
3	Do the people close to you know your HIV status?		
4	If you choose to formula feed, would you be comfortable giving infant formula in public/community?		
5	Can you prepare formula feed and feed your baby every 2-4 hrs (6-12x/day) day and night for up to six months?		
6	Do you have anybody that could help you to prepare and feed your baby whenever you are not available?		
7	Do you have a source of clean water or tap water in your home/compound or close by?		
8	Do you have a refrigerator in your home?		
9	Do you have constant supply of electricity?		
10	Would be able to ensure constant supply of formula milk?		
11	When needed, will you be able to buy formula milk in a neighbourhood shop?		
12	Can you afford $... (Amount varies for different countries) on infant formula for at least six months without affecting the health and nutrition of other family members?		
13	Can you afford $... (Amount varies for different countries) on utensils and cooking fuel required for preparing infant formula for at least six months?		
14	Do you have a source of regular income?		
15	Does the father of your child have a source of regular income?		
16	Would you always remember to wash your hands with clean water after using the toilet and before preparing formula feeds		
17	Would you be able to wash with clean water all utensils required for preparing formula feeds?		
18	Is there a health facility that provides Maternal and Child health services that you can go to if your baby need medical attention?		
TOTAL NUMBER OF YES RESPONSES			

**FEEDING OPTIONS**	
	**≥15; Formula feed**
	**10-14; Indecisive, further counselling and questions**
	**<10; Breastfeed**
